# Genetic Evidence for Genotoxic Effect of Entecavir, an Anti-Hepatitis B Virus Nucleotide Analog

**DOI:** 10.1371/journal.pone.0147440

**Published:** 2016-01-22

**Authors:** Lei Jiang, Xiaohua Wu, Fang He, Ying Liu, Xiaoqing Hu, Shunichi Takeda, Yong Qing

**Affiliations:** 1 Department of Pharmacology, West China School of Pharmacy, Sichuan University, Chengdu, Sichuan, 610041, China; 2 Regenerative Medicine Research Center, and State Key Laboratory of Biotherapy and Cancer Center, West China Hospital, Sichuan University, Chengdu, Sichuan, 610041, China; 3 Center of Infectious Diseases, Division of Molecular Biology of Infectious Diseases, State Key Laboratory of Biotherapy, West China Hospital, Sichuan University, Chengdu, Sichuan, 610041, China; 4 Department of Radiation Genetics, Graduate School of Medicine, Kyoto University, Kyoto, 606–8501, Japan; University of Massachusetts Medical School, UNITED STATES

## Abstract

Nucleoside analogues (NAs) have been the most frequently used treatment option for chronic hepatitis B patients. However, they may have genotoxic potentials due to their interference with nucleic acid metabolism. Entecavir, a deoxyguanosine analog, is one of the most widely used oral antiviral NAs against hepatitis B virus. It has reported that entecavir gave positive responses in both genotoxicity and carcinogenicity assays. However the genotoxic mechanism of entecavir remains elusive. To evaluate the genotoxic mechanisms, we analyzed the effect of entecavir on a panel of chicken DT40 B-lymphocyte isogenic mutant cell line deficient in DNA repair and damage tolerance pathways. Our results showed that *Parp1*^*-/-*^ mutant cells defective in single-strand break (SSB) repair were the most sensitive to entecavir. *Brca1*^*-/-*^, *Ubc13*^*-/-*^ and translesion-DNA-synthesis deficient cells including *Rad18*^*-/-*^ and *Rev3*^*-/-*^ were hypersensitive to entecavir. *XPA*^*-/-*^ mutant deficient in nucleotide excision repair was also slightly sensitive to entecavir. γ-H2AX foci forming assay confirmed the existence of DNA damage by entecavir in *Parp1*^*-/-*^, *Rad18*^*-/-*^ and *Brca1*^*-/-*^ mutants. Karyotype assay further showed entecavir-induced chromosomal aberrations, especially the chromosome gaps in *Parp1*^*-/-*^, *Brca1*^*-/-*^, *Rad18*^*-/-*^ and *Rev3*^*-/-*^ cells when compared with *wild-type* cells. These genetic comprehensive studies clearly identified the genotoxic potentials of entecavir and suggested that SSB and postreplication repair pathways may suppress entecavir-induced genotoxicity.

## Introduction

Chronic infection with hepatitis B virus (HBV) remains a major global health problem. Currently, the number of persons infected with HBV is approximately 2 billion, and over 400 million are suffering from chronic hepatitis B (CHB) worldwide [1]. Nucleoside analogues (NAs) have been the most frequently used treatment option for CHB patients due to their effects on inhibiting replication of hepatitis B virus [2]. The majority of CHB patients need long-term treatment with NAs [3, 4]. Entecavir, a carbocyclic 2’-deoxyguanosine analog, possesses potent and selective anti-hepatitis B virus (anti-HBV) activity. Entecavir induces a rapid biochemical and virologic response in CHB patients and has a high genetic barrier to resistance [5, 6]. These characteristics make it recommended as a first-line antiviral therapy for patients with CHB by international guidelines [7–9]. Unfortunately, the US prescribing information sheet and European centralized procedure (CP) indicate that entecavir is carcinogenic in primary human lymphocytes and induces lung, vascular, brain, liver and skin tumors in mice and rats [10–12]. Recently, Brown et al. reported that entecavir can be incorporated and embedded into the human genome via primer extension or subsequent ligation and that may contribute to a putative mechanism of carcinogenicity [13]. However, further studies remain to be done to gain a better understanding of the genotoxicity mechanisms of entecavir.

DNA damage occurs daily with physical and chemical mutagens. In response to it, cells have evolved specific method of repairing the damages, including base excision repair (BER), nucleotide excision repair (NER), single-strand break (SSB) repair and double-strand break repairs consist of nonhomologous end joining (NHEJ) repair and homologous recombination (HR) [14]. DNA lesions that remain unrepaired before entering S phase often cause the collapse of DNA replication, leading to chromosomal breaks in mitotic cells and subsequent cell death [15]. To restart blocked DNA replication forks, cells have evolved postreplication repair (PRR), including HR and translesion DNA synthesis (TLS) pathways [16]. TLS pathways release the replication block by filling a daughter strand gap, employing a number of DNA polymerases, including *Rad18*, *Rad6* and *Polζ* [17], whereas HR relies on recombination processes [18]. Both of *Brca1* and *Ubc13* play critical role in PRR [19, 20].

DT40 cells have been a favorable tool for studying the DNA repair pathways due to its high-frequency gene targeting [21, 22]. Previously, we had generated a panel of DNA-repair deficient DT40 clones which were defective in BER, NER, SSBR, NHEJ, HR and TLS respectively (Table 1). Due to the defective function on DNA repairs, these mutant clones are also sensitive to different genotoxic chemicals [23–25]. The characteristics of DNA-repair deficient DT40 clones are advantageous and useful for investigating the mechanisms of chemical genotoxicity [15, 26]. In this study, to explore the underlying mechanisms that suppress entecavir-induced genotoxicity, we performed comprehensive analyses of the genotoxicity with a panel of DT40 DNA repair mutants (Table 1).

**Table 1:** DNA repair genes mutated in the analyzed DT40 clones.

Gene	Function	Reference
*Rev3*	TLS, HR (catalytic subunit of Polξ)	[27]
*XPA*	An initial step of nucleotide excision repair	[28]
*Ubc13*	Ubc13 is related to the initial step of HR and	[29, 30]
	postreplication repair	
*Parp1*	Poly(ADP) ribosylation, related to	[31]
	single-strand break and base excision repair	
*Brca1*	HR	[32]
*Brca2*	HR	[33]
*Rad18*	TLS	[28]
*Polβ*	Base excision repair	[34]
*Fen1*	Base excision repair, processing 5’flap in	[35]
	long-patch and lagging strand DNA	
	replication	
*Xrcc2*	Rad51 paralog, homologous recombination,	[25]
	promotion of Rad51 assembly	
*CtIP(S332A*^*−/−*^*)*	Eliminating covalently bound polypeptides	[36]
	from DSBs	
*Ku70*	Initial step for NHEJ dependent DSB repair	[37]

DSB, double-strand break; HR, homologous recombination; NHEJ, nonhomologous end joining repair; TLS, translesion DNA synthesis

## Results

### Mutant cells defective in DNA repair pathways were sensitive to entecavir

**Fig 1:**
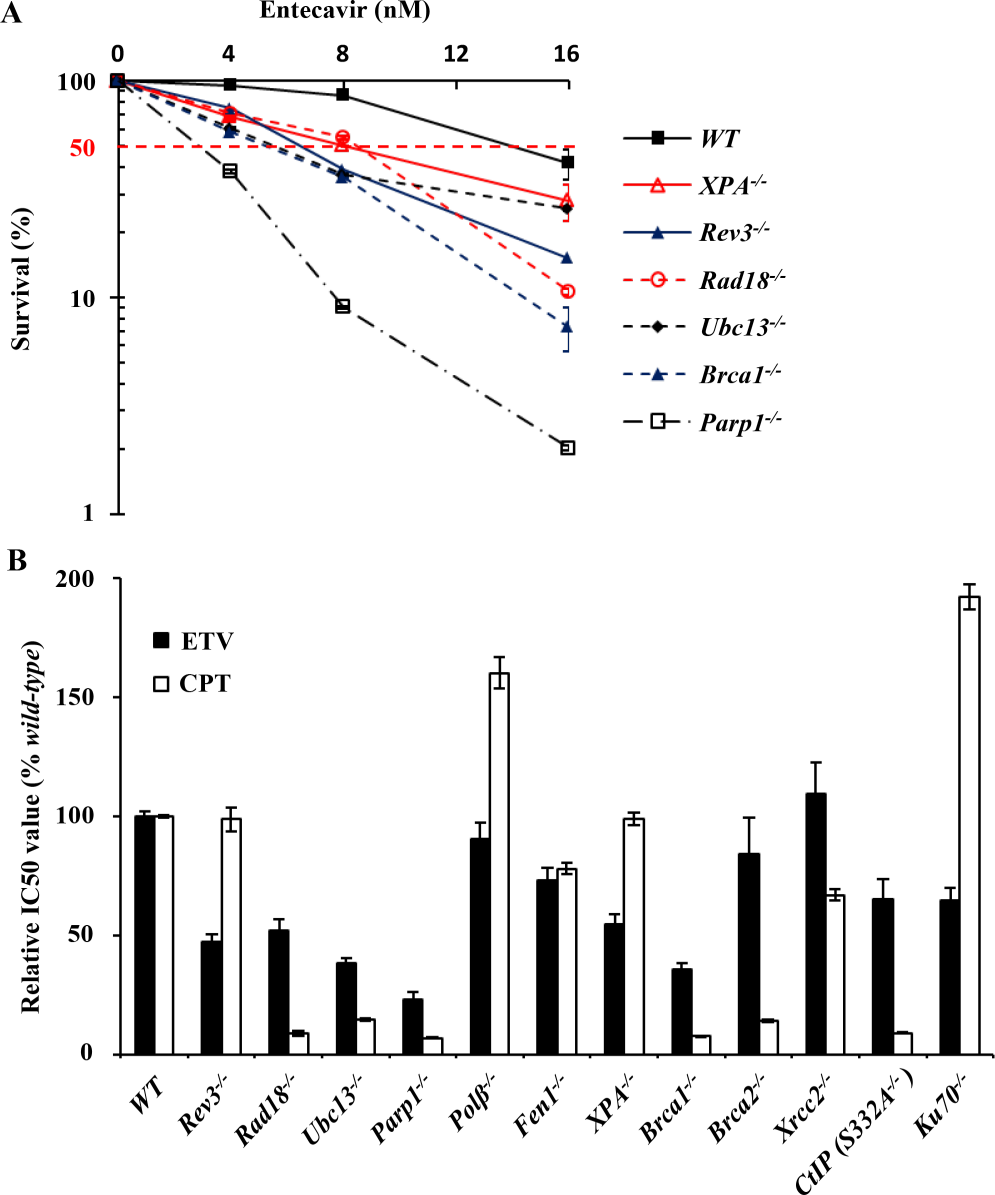
Mutant cells defective in DNA repair pathways were sensitive to entecavir. (A) The X-axis represents the concentration of entecavir and the Y-axis represents the relative number of surviving cells at 72 hours. Survival data were log-transformed giving approximate normality. Analysis of covariance (ANCOVA) was used to test for differences in the linear dose-response curves between *wild-type* and a series of mutant cells. A p-value < 0.05 was considered to be significant. (B) Relative IC50 values of cell survival results in *wild-type* and their mutants exposed to entecavir or CPT. Each IC50 value was calculated from results of cell survival data shown in Fig 1A and S1 Fig Relative IC50 values were normalized according to the IC50 value of parental *wild-type* cells. The IC50 was calculated by SPSS software version13.0. Data shown are the means of three experiments. Values shown are mean ± SD.

To study the genotoxicity of entecavir, we evaluated the effects of entecavir on a panel of gene disrupted clones (Table 1) by MTT assay. Camptothecin (CPT), a topoisomerase I poison, was selected as a positive control. We continuously exposed *WT* and mutant cells to entecavir or CPT at various concentrations for 72h. The results indicated that entecavir inhibited the growth of DT40 cells in a dose-dependent manner. As shown in Fig 1, *Parp1*^*-/-*^ cells defective in DNA SSB exhibited the hypersensitivity to entecavir. *Ubc13* deficient cells and TLS-deficient clones, both *Rad18*^*-/-*^ and *Rev3*^*-/-*^, were sensitive to entecavir. To investigate the two major double-strand break repair pathways, HR and NHEJ, *Brca1*^*-/-*^, *Brca2*^*-/-*^, *Xrcc2*^*-/-*^ and *Ku70*^*-/-*^ were analyzed. Only *Brca1*^*-/-*^ cells manifested significant sensitivity to entecavir. *Xrcc2*^*-/-*^ cells were even slightly resistant to entecavir. The other DNA repair gene deficient cells, including *XPA*^*-/-*^ cells were also sensitive to entecavir, but *Polβ*^*-/-*^, *Fen1*^*-/-*^ and *CtIP (S332A*^*-/-*^*)* cells were not. CPT can induce DNA damage by inhibiting the ligation of SSBs that are formed during the normal functioning of topoisomerase I [38]. Unrepaired SSBs are converted to double-strand breaks upon replication. It has been shown that CPT induced double-strand breaks are mainly repaired by HR in DT40 cells [39]. As shown in Fig 1B and S1 Fig, *Parp1*^*-/-*^, *Rad18*^*-/-*^, *Ubc13*^*-/-*^, *CtIP (S332A*^*-/-*^*)*, *Brca1*^*-/-*^ and *Brca2*^*-/-*^ cells were hypersensitive to CPT. In contrast, *Polβ*^*-/-*^ and *Ku70*^*-/-*^ were resistant to CPT, as previously reported [39]. This observation indicated that entecavir may exert potential genotoxic mechanisms which mainly associate with SSB repair and PRR, but not a double-strand break repair.

### Entecavir induced the accumulation of γ-H2AX in nuclei of DT40 cells

**Fig 2:**
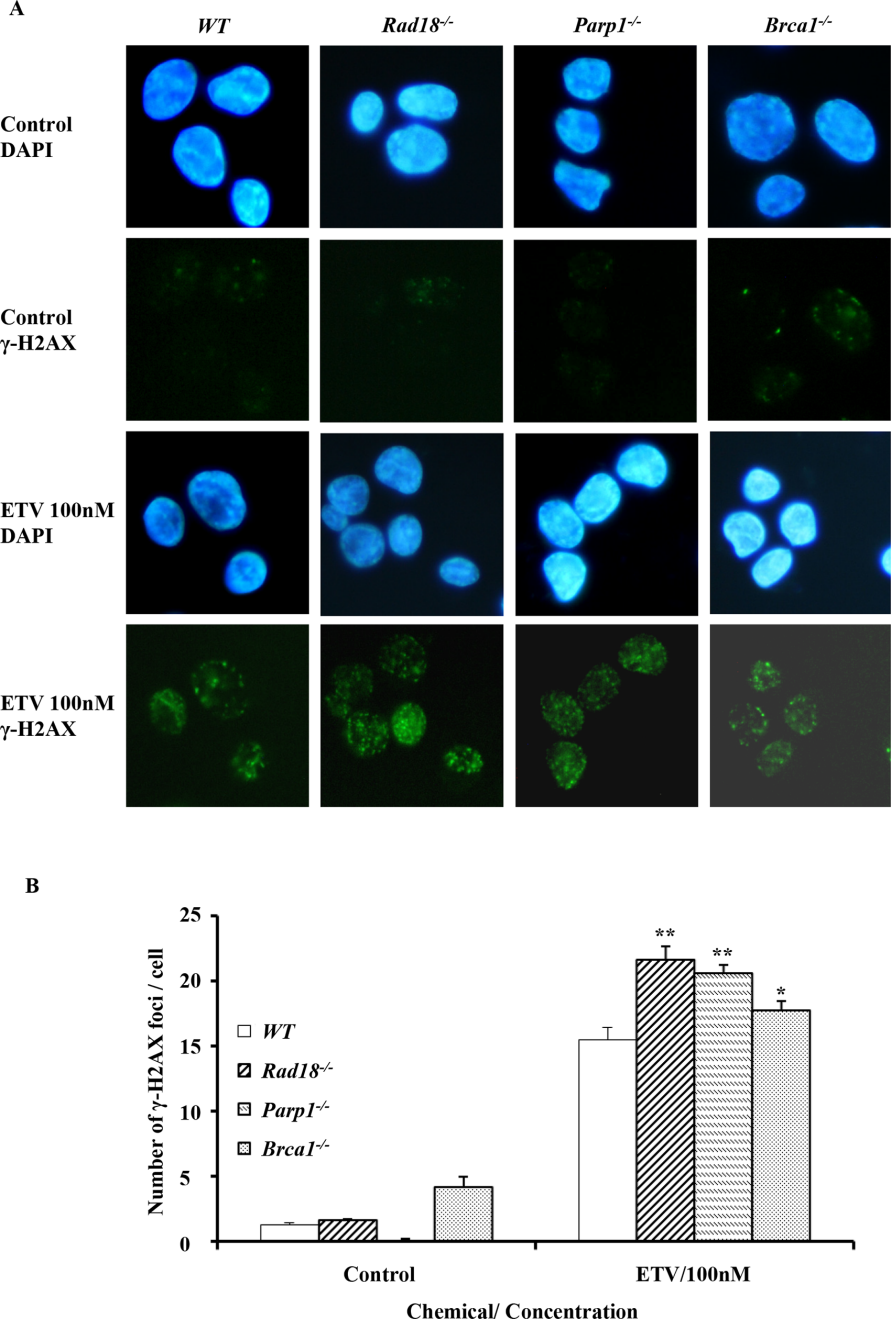
Entecavir induced the accumulation of γ-H2AX in nuclei of DT40 cells. (A) Immuno-staining of wild-type (*WT*) and mutant DT40 clones using anti-γ-H2AX antibody and DAPI. Cells were fixed 6 hours after treated with entecavir 100nM. ETV, entecavir. (B) Quantification of γ-H2AX foci in individual cells of the indicated genotype. Cells were treated with entecavir 100nM for 6h. Data shown are the means of three experiments. Values shown are mean ± SD. ** P < 0.01, * P < 0.05 compared to *WT*. More than 100 cells were analyzed for each data point.

To investigate entecavir-induced damages responses, we determined the number of γ-H2AX foci, a sensitive molecular marker of DNA damage in nuclear DNA [40]. The immunofluorescence assay was conducted using *WT*, *Parp1*^*-/-*^, *Rad18*^*-/-*^ and *Brca1*^*-/-*^ cells for entecavir. Six hours after exposure to 100nM entecavir, *Parp1*^*-/-*^, *Rad18*^*-/-*^ and *Brca1*^*-/-*^ exhibit more numbers of γ-H2AX foci when compared with *WT* cells (Fig 2A and 2B). The increased accumulation of γ-H2AX in nuclei of *Parp1*^*-/-*^, *Rad18*^*-/-*^ and *Brca1*^*-/-*^ cells suggested increased DNA damages, which is consistent with hypersensitivity of these cells to entecavir.

### DNA repair-deficient cells showed a marked increase in entecavir-induced chromosome breaks

**Fig 3:**
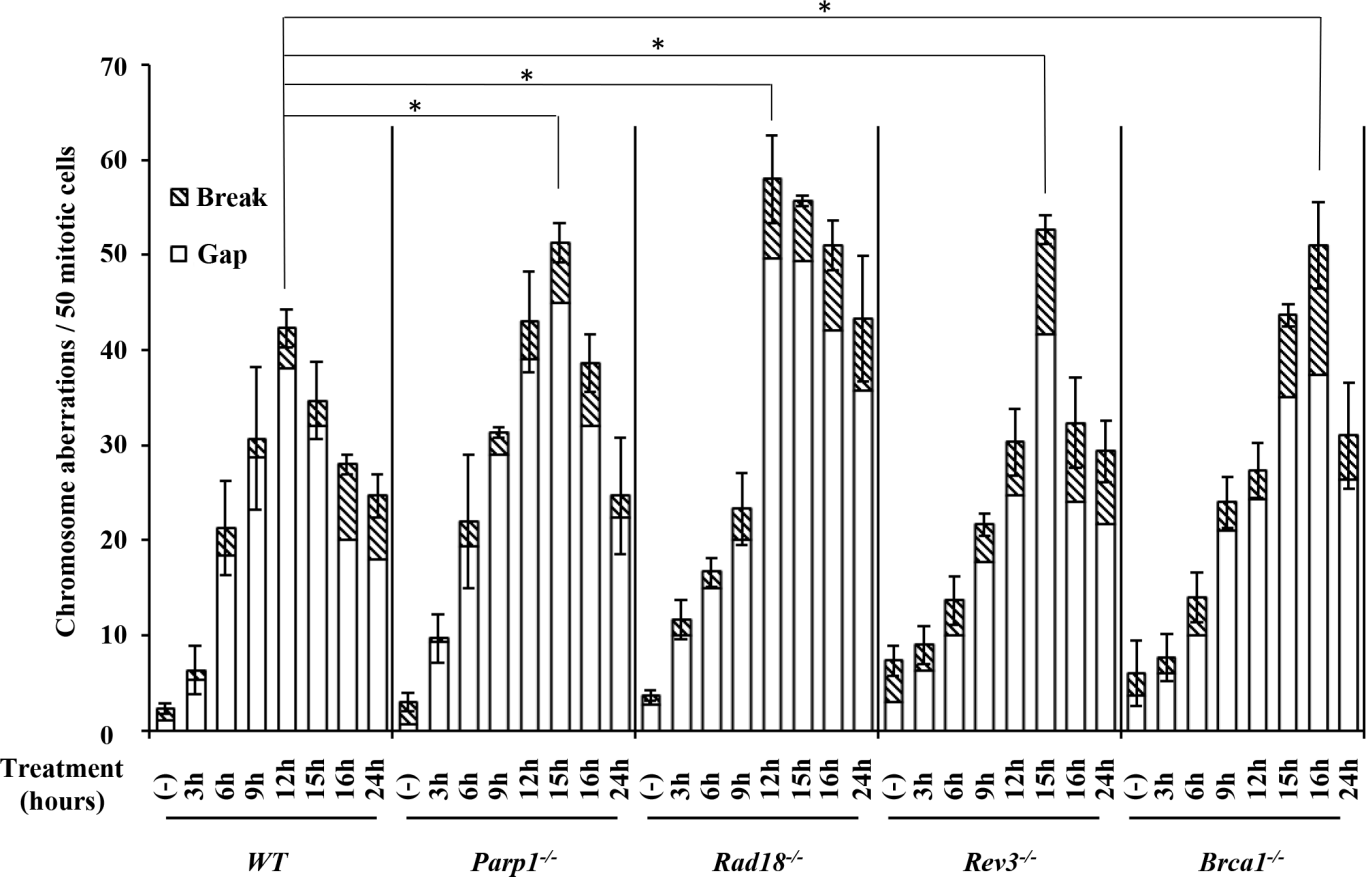
DNA repair-deficient cells showed a marked increase in entecavir-induced chromosome breaks. Increased frequency of chromosomal aberrations (CAs) in DNA repair-deficient cells and *WT* treated with entecavir (200nM) from 3 hours to 24 hours. Data are derived from 50 metaphase cells for each treatment. The experiments were independently repeated three times for statistical analysis. Values shown are mean ± SD. * P < 0.05 compared to WT. The differences between the *WT* and DNA repair-deficient cell lines were tested for statistical significance using t-test.

**Fig 4:**
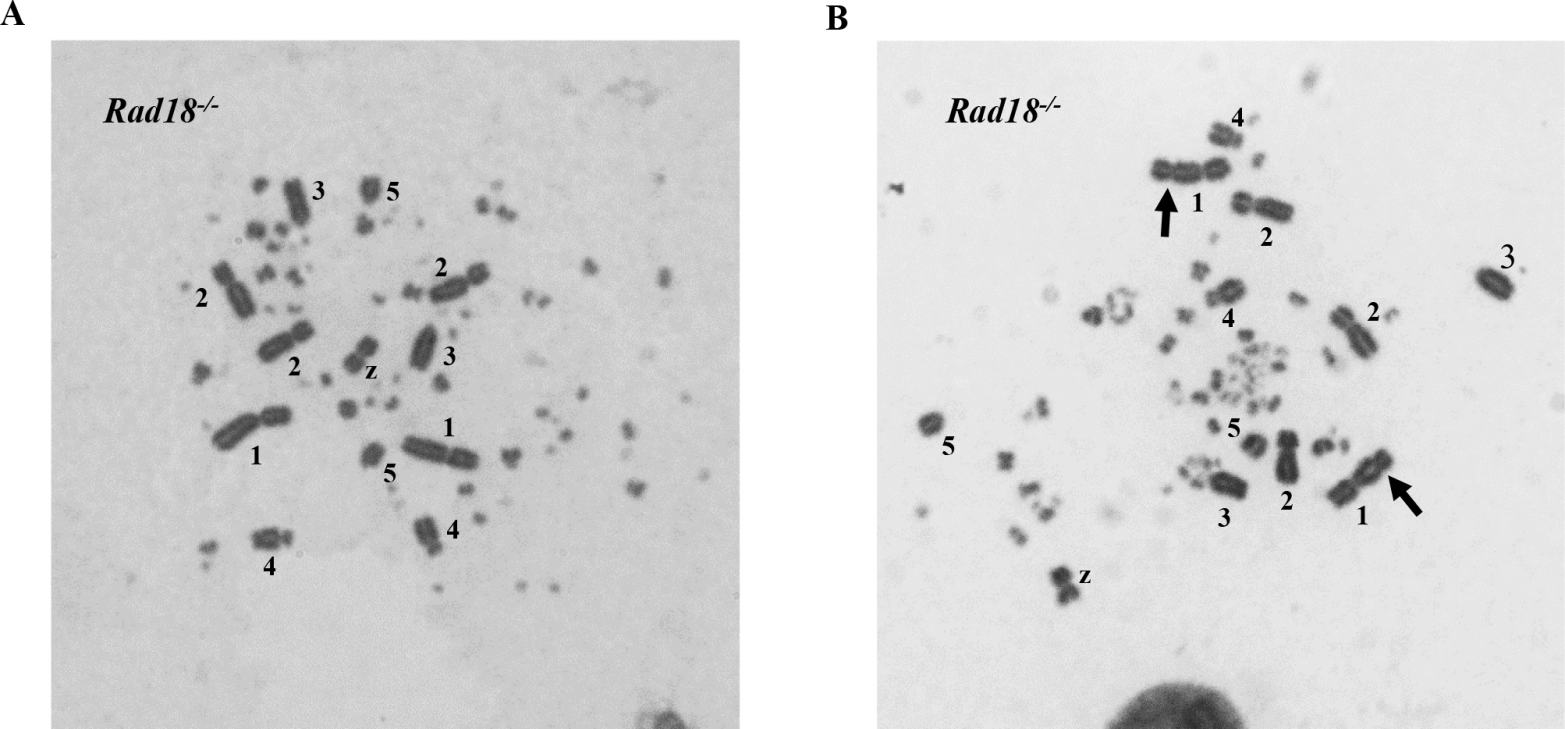
Representative karyotype analysis of entecavir pretreated *Rad18*^*-/-*^ cells. (A) Representative karyotype of untreated *Rad18*^*-/-*^ cells. (B) Chromosomal aberrations (CAs) in *Rad18*^*-/-*^ cells following 200nM entecavir pretreatment for 15 h. Macrochromosomes 1–5 and Z are identified. Chromosome gaps are shown by arrow.

To further investigate entecavir-induced DNA damages, we measured cytologically detectable chromosomal aberration in chromosome spreads. *WT*, *Parp1*^*-/-*^, *Rad18*^*-/-*^, *Brca1*^*-/-*^ and *Rev3*^*-/-*^ cells were exposed to entecavir 200nM from 3 to 24 hours (Figs 3 and 4). Interestingly, *WT*, *Rad18*^*-/-*^, *Rev3*^*-/-*^, *Parp1*^*-/-*^ and *Brca1*^*-/-*^ cells demonstrated a monophasic pattern of induced chromosome breaks; the peaks were detectable at 12, 12, 15, 15 and 16 hours respectively (Fig 3). The peaks were significantly higher in DNA repair-deficient cells than in *WT* cells. Remarkably, the number of chromosome gap was higher than that of chromosome break in both *WT* and DNA repair-deficient cells. Entecavir mainly induced chromosome gap, but not break in metaphase, further suggesting its action for SSB, but not double-strand break. The increased chromosomal aberrations in *Parp1*^*-/-*^, *Brca1*^*-/-*^, *Rad18*^*-/-*^ and *Rev3*^*-/-*^ when compared with *WT* just reflected these genes have critical role in preventing entecavir-induced chromosomal aberrations.

## Discussion

Entecavir, a carbocyclic 2’-deoxyguanosine analog, was widely used for HBV clinical therapies by inhibiting the HBV polymerase, competing with dGTP. In this study, we used the concentration of entecavir from 4 to 64 nM, which was based upon the maximal clinical exposure concentration 30nM [41, 42], to analyze the sensitivity of a panel of DNA repair deficient DT40 cells to entecavir. These cells include SSB repair mutant *Parp1*^*-/-*^, BER repair mutant *Polβ*^*-/-*^, NER mutant *XPA*^*-/-*^, HR repair mutants *Brca1*^*-/-*^, *Brca2*^*-/-*^, *Xrcc2*^*-/-*^ and *CtIP (S332A*^*-/-*^*)*, NHEJ repair mutant *Ku70*^*-/-*^, PRR mutants *Ubc13*^*-/-*^, *Rad18*^*-/-*^ and *Rev3*^*-/-*^ as well as flap structure-specific endonuclease 1 mutant *Fen1*^*-/-*^. Results showed that the SSB repair mutant of *Parp1*^*-/-*^, PRR mutants *Rad18*^*-/-*^, *Rev3*^*-/-*^, *Ubc13*^*-/-*^ and *Brca1*^*-/- *^cells were significant sensitive to entecavir. At the same time, we found that the sensitivities of *Parp1*^*-/-*^, *Rad18*^*-/-*^, *Ubc13*^*-/-*^ and *Brca1*^*-/-*^ cells to entecavir were similar to CPT. In contrast, *Brca2*^*-/-*^ and *CtIP (S332A*^*-/-*^*)* were hypersensitive to CPT, not entecavir. Further immunofluorescent analysis indicated that the number of γ-H2AX foci was significantly increased in SSB repair mutant *Parp1*^*-/-*^ and TLS mutant *Rad18*^*-/-*^ cells. Chromosomal aberration assay also proved that the number of chromosome gap was significantly increased in SSB repair mutant *Parp1*^*-/-*^ and PRR mutants, *Brca1*^*-/-*^, *Rad18*^*-/-*^ and *Rev3*^*-/-*^ compared with *WT*. The data strongly suggest that entecavir is genotoxic and two DNA repair pathways, SSB repair and PRR, are responsive to suppress the genotoxicity.

SSBs in DNA are often raised by loss of a single nucleotide and by damaged 5’- and / or 3’-termini at the site of the break [43]. A multitude of factors trigger SSBs. Erroneous incorporation of ribonucleotides into DNA is the commonest sources of endogenous SSBs [44]. *Parp1* is a sensor protein, which plays an important role in DNA SSB detection [43, 45]. In the current study, we found that *Parp1*^*-/-*^ cells exhibited the hypersensitive to entecavir and manifested significantly increase in the number of γ-H2AX foci and chromosomal aberrations compared with *WT*, suggesting that entecavir may induce SSBs. As *Parp1* also functions in BER, we examined the sensitivity of BER deficient cells *Polβ*^*-/-*^, and results showed *Polβ*^*-/-*^ cells were not significantly sensitive to entecavir. But we found the NER deficient cells *XPA*^*-/-*^ were slightly sensitive to entecavir.

We also examined *Brca1*^*-/-*^, *Brca2*^*-/-*^, *Xrcc2*^*-/-*^, *CtIP(S332A*^*-/-*^*)* and *Ku70*^*-/-*^ cells, which respectively defective in HR and NHEJ, two major pathways for double strand breaks repair [37], and only *Brca1*^*-/-*^ cells showed sensitivity to entecavir. We speculate that double strand breaks might not be the majority of entecavir-induced DNA damages. Recent studies had proved that besides the function on HR for double strand breaks repair, *Brca1* could directly recruits translesion polymerases, such as *Polη* and *Rev1*, to the lesions through protein-protein interactions, suggesting its critical role in PRR [19]. Currently, we found *Rev3*^*-/-*^ and *Rad18*^*-/-*^ were also sensitive to entecavir and had increased entecavir-induced chromosomal aberrations (Fig 3). Both *Rad18* and *Rev3* play critical role in PRR pathway. Studies indicated that *Rad18* forms a complex with *Rad6* to promote PCNA mono-ubiquitination, which is a crucial step in PRR pathway [16], whereas *Rev3* gene encodes the catalytic subunit of DNA *Polξ*, which is involved in TLS, one of PRR pathway [27]. Furthermore, *Ubc13*, a K63-linked E2 Ub-conjugating enzyme, have been proved to function on both HR and error-free PRR [29, 20]. Results showed cells deficient in *Ubc13* were also sensitive to entecavir. Above all, we hypothesize that entecavir induces DNA damage, which may collapse the replication forks and PRR pathway might release the replication fork stall.

**Fig 5:**
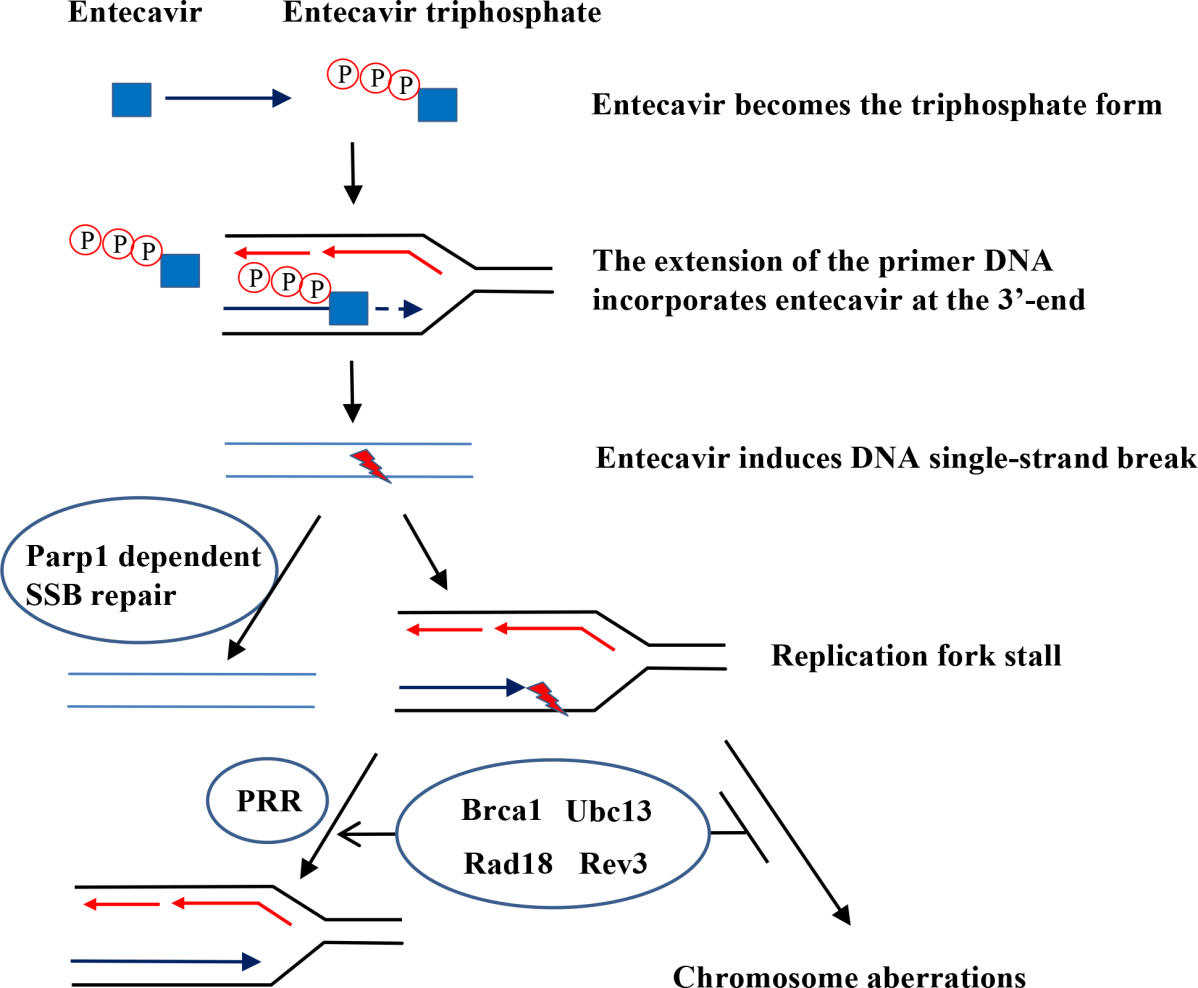
Model of entecavir-induced genotoxicity related to single-strand break (SSB) repair and postreplication repair (PRR) pathway. The triphosphate of entecavir is incorporated into DNA strand by host replication or repair polymerases, which blocking extension of the nascent strand and inducing DNA SSB and *Parp1* dependent repair. The entecavir-induced DNA lesions could also be repaired by PRR to avoid the replication fork collapse and chromosomal breaks when cells enter into S phase.

Entecavir was metabolized by phosphorylation to triphosphate (TP) form in mammalian cells by cellular enzymes to inhibit HBV DNA replication [46]. The mechanism for chain termination by entecavir is likely to involve incorporation and abortive extension of ETV-containing HBV DNA [47]. Some studies reported that entecavir displays no interaction with host polymerase and failed to be incorporated into human DNA [41]. Nonetheless, the recent study by Brown et al. showed that entecavir can be incorporated and embedded into the human genome via primer extension with human X or Y polymerases or subsequent ligation [13]. One possible model that could explain our data is shown in Fig 5. The triphosphate of entecavir is incorporated into DNA strand by host replication or repair polymerases, which blocking extension of the nascent strand and inducing DNA SSB and *Parp1* dependent repair. The entecavir-induced DNA lesions could also be repaired by PRR to avoid the replication fork collapse and chromosomal breaks when cells enter into S phase.

NAs have been shown effective inhibition of HBV replication, which delay the progression of liver cirrhosis, reduce the incidence of HBV related liver cancer, above all, increase the life span of the patients [2]. Until now, most current guidelines recommended that a long-term treatment with NAs is essential to majority CHB patients, even a life-long therapy for CHB with cirrhosis. And entecavir is one of the first-line therapies. Especially in those with decompensated liver disease, undergoing immunosuppressive treatment or with contraindications, and those unwilling to receive Peg-IFN, entecavir or tenofovir is the only therapeutic options in patients [4]. However, long-term safety data are still lacking for NAs, including entecavir [3, 7]. Some studies demonstrated entecavir was clastogeic at 36μM in primary human lymphocytes [10]. Considering that entecavir inhibited HBV DNA synthesis in the nanomolar range [42], so they thought it’s safe to humans. But in our study, entecavir induced DNA damage at nanomolar in DT40 cells, especially in the more sensitive DNA repair deficient cells. So we think it is necessary to monitor the genotoxicity of NAs, especially entecavir, and to restrict treatment period.

Much work remains to be done to gain a better understanding of the mechanism of genotoxicity of entecavir. A better understanding of entecavir-induced genotoxicity may contribute to development of new drugs for the treatment or prevention of chronic hepatitis B with higher therapeutic efficacy and less genotoxicity.

## Materials and Methods

### Chemicals

Entecavir was obtained from Sigma-Aldrich (St. Louis, MO, USA). CPT was purchased from Shanghai standard Biotech Co., Ltd. Stock solution of entecavir (100 μM) and CPT (100 μM) were prepared in dimethyl sulfoxide (DMSO) and stored at -20°C in aliquots until use. Pharmaceuticals were dissolved using DMSO and maximum volume of the solvent did not exceed 0.1% of the culture medium.

### Cell lines and cell culture

Cell lines used in this study are listed in Table 1. Cells were cultured as described before [37]. The DT40 cell lines were cultured in RPMI-1640 (Gibco) supplemented with 10% heat-inactivated fetal bovine serum, 1% chicken serum, 1% penicillin streptomycin (Gibco) and 50 μM β-mercaptoethanol (Gibco) at 39.5°C in a humidified atmosphere of 5% CO_2_ (Sanyo, Osaka, Japan).

### MTT assay

The cytotoxicity of entecavir or CPT on DT40 cell lines was determined by the MTT assay [48, 49]. Cells were seeded in 96-well plates (Costar Corning, Rochester, NY). Cells were treated with entecavir or CPT at various concentrations, and carrier DMSO (< 0.1%) was used as a control, 3 wells were included in each concentration. After 72h, the cells were treated with 20 μl of 5 mg/ml MTT (Amresco, USA) and the resulting formazan crystals were dissolved in 50 μl of 20% SDS (pH4.7) over night. The absorbance at 570 nm was measured using wells without cells as blanks. All experiments were performed in triplicate. The 50% inhibiting concentration (IC50) was calculated by SPSS softwareversion13.0.

### Chromosomal aberrations analysis

Karyotype analysis was done as previously described [50]. Briefly, cells were treated with entecavir in the complete medium. To arrest cells in metaphase, 0.1% colcemid (GIBCO-BRL, Grand Island, NY, USA) was added 3h before harvest. Then, cells were resuspended in 1 ml of 75 mM KCl for 15 min at room temperature, and fixed in 5 ml of Carnoy's solution (mixture of methanol and acetic acid, 3:1). The cells suspension was dropped onto ethanol-cleaned slides and dried by a flame. The slides were stained with 5% Giemsa solution for 7 min, and dried after being rinsed carefully with water. The chromosomal aberrations were observed under a light microscope (with 1000× magnification). All experiments were performed in triplicate. Data are derived from 50 metaphase cells for each treatment. The scoring criteria were essentially the same as those of ISCN [51]. According to ISCN, a break is defined as a discontinuity of a chromosome that shows a clear misalignment of the distal fragment of a broken chromosome. A gap is defined as a clear non-staining region on a chromosome [50]. Chromosome gaps and breaks were both sister chromatids of a single chromosome broken at the same locus, whereas chromatid gaps and breaks were a single chromatid broken.

### Immunofluorescent

Experimental condition for immunofluorescent analysis is described previously [52]. Briefly, DT40 cells (10^5^ cells) were harvested on a slide glass after treated with entecavir for different hours. Cells were fixed with 3% formaldehyde for 10 min at room temperature and then washed with PBS. For permeabilisation, cells were incubated with 0.1% NP-40 for 15 min at room temperature and washed again with PBS. After blocking with 3% BSA, fixed cells were treated with specific antibodies. The cells were incubated with anti-phospho-Histone H2AX (Ser139) mouse monoclonal antibody at a dilution of 1:500 (Millipore, Billerica, MA, USA). Following another washing step with PBS, cells were incubated for 1h with a secondary Alexa Fluor 488-conjugatedanti-mouse antibody (1:1000; Beyotime, Wuhan, China).

## Supporting Information

S1 Fig Sensitivity of *wild-type* (*WT*) and isogenic DNA-repair deficient DT40 clones to entecavir or CPT. Cellular sensitivities to entecavir (A) or CPT (B and C) were analyzed using the same method as in Fig 1.
(TIF)
