# Supplementary figures and images for: Genetic Evidence for Genotoxic Effect of Entecavir, an Anti-Hepatitis B Virus Nucleotide Analog
Source: PLoS One. 2016 Jan 22;11(1):e0147440. doi: 10.1371/journal.pone.0147440 (PMC4723259; doi:10.1371/journal.pone.0147440)

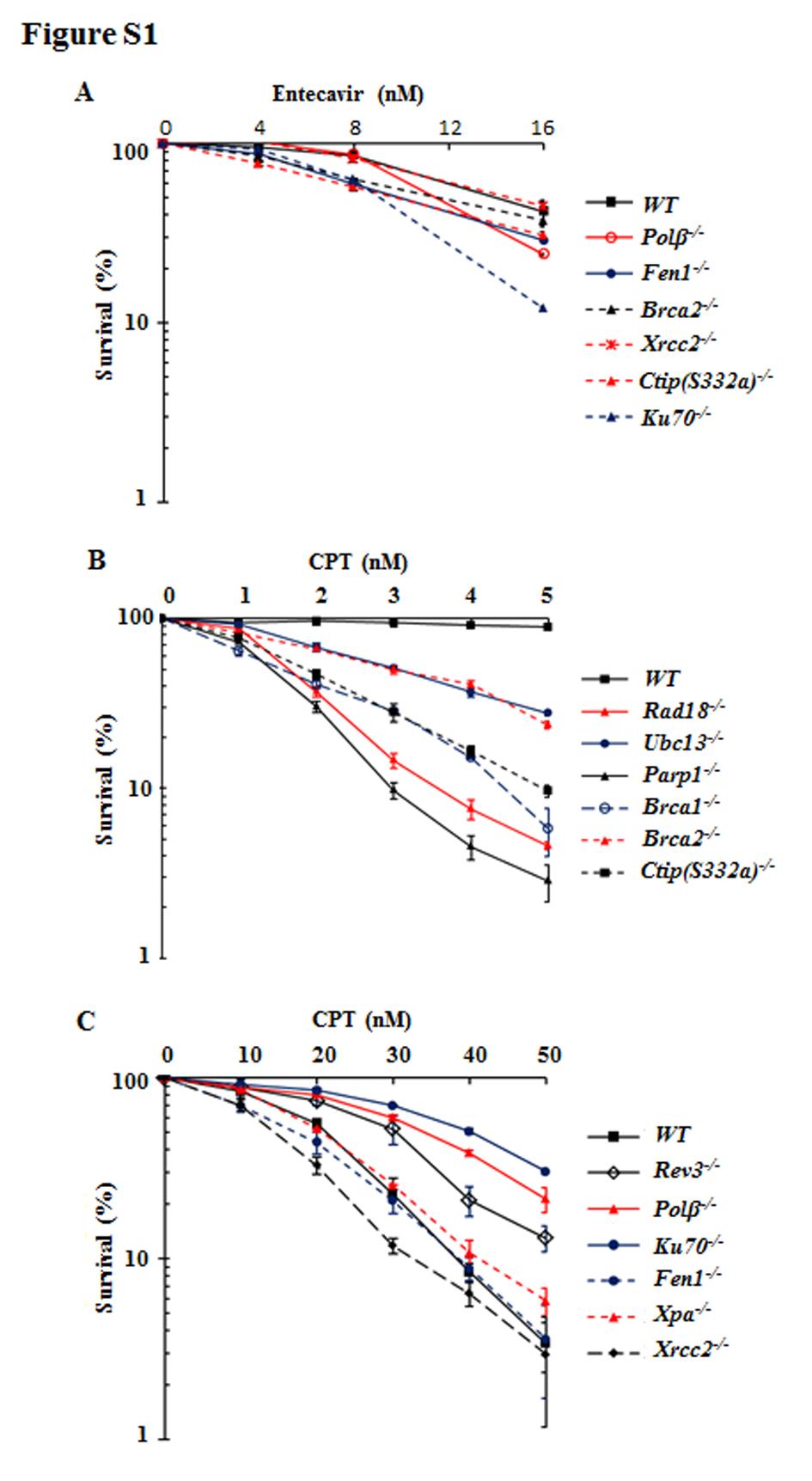

Supplement: S1 Fig — Cellular sensitivities to entecavir (A) or CPT (B and C) were analyzed using the same method as in Fig 1. (TIF) [file pone.0147440.s001.tif]
